# Beyond Melanin: A Literature Review of Photoprotection Mechanisms and Gaps in Skin of Color

**DOI:** 10.7759/cureus.113666

**Published:** 2026-07-30

**Authors:** Pamella Leybengrub, Wintana Eyob, Lina Petrossian, Saumya Shetty, Berkeley A Scott, Mohamad Bilal Kebbe, Karen Vo

**Affiliations:** 1 Renaissance School of Medicine, Stony Brook University, Stony Brook, USA; 2 College of Medicine, University of Cincinnati, Cincinnati, USA; 3 School of Medicine, California University of Science and Medicine, Colton, USA; 4 School of Osteopathic Medicine, Rowan-Virtua School of Osteopathic Medicine, Stratford, USA; 5 School of Medicine, University College Dublin, Dublin, IRL; 6 Department of Dermatology, Istanbul Okan University, Istanbul, TUR; 7 Transitional Year Residency Program, Desert Regional Medical Center, Palm Springs, USA

**Keywords:** health disparities, photoaging, photoprotection, pigmentary disorders, skin of color, sunscreen, visible light

## Abstract

There is a critical need for tailored photoprotection in skin of color (SOC) populations. SOC has higher eumelanin-to-phenomelanin ratios, more mature melanosomes, and enhanced DNA repair capacity compared with lighter skin. The quantity and distribution of melanin in darker skin provide natural photoprotection. This leads to a misconception that SOC populations are inherently protected from all photodamage and serves as a barrier to proper photoprotection for these populations. Although the skin cancer incidence is lower in dark skin, mortality is higher, perhaps as a result of late diagnosis or misdiagnosis. These populations are still susceptible to skin damage from ultraviolet (UV) radiation, resulting in exacerbation of various dermatologic conditions, including pigmentation disorders, photodermatoses, and skin cancer.

This review aims to comprehensively examine the photoprotection needs for SOC populations, addressing gaps between physiological characteristics and clinical outcomes, evaluating current photoprotection practices, examining educational implications, and considering future directions for photoprotection for SOC. A literature search was performed using PubMed and Google Scholar. The search was conducted from database inception through July 15, 2026. Articles were included if they addressed photoprotection, photodamage, photoaging, pigmentary disorders, photodermatoses, or skin cancer in SOC populations.

Visible light (VL) and long-wave UVA1 are significant contributors to pigmentary alteration in SOC. The major drivers of pigmentation in SOC and photodamage can manifest as pigmentary disorders, particularly melasma and post-inflammatory hyperpigmentation (PIH), photoaging, photodermatoses, and skin cancer risk. Comprehensive photoprotection strategies for individuals with SOC include behavioral sun avoidance measures, sun-protective clothing, avoiding harmful behaviors, and community-based education programs. Sunscreen formulations for SOC should be tailored to skin phototype (SPT), with an SPF of 30 and broad protection that covers UVA, VL, and infrared A to limit hyperpigmentation and photoaging. Iron oxide-containing sunscreens provide VL protection and reduce white cast. Niacinamide-containing sunscreens are helpful for reducing PIH. Since UV and VL generate reactive oxygen species (ROS) that promote inflammation and persistent PIH, antioxidants in sunscreen are important in SOC to neutralize this damage and support DNA repair.

Comprehensive photoprotection is essential for SOC populations. SOC individuals face unique manifestations of photodamage. Effective photoprotection in SOC requires extension beyond traditional UVB protection to meet the specific needs of SOC. Additionally, targeted education, community engagement, and barriers to care need to be addressed to improve outcomes. It is important for the dermatology community to commit to inclusivity and advocate for equitable access to photoprotection resources for all.

## Introduction and background

As racial diversity increases, there is a critical need for tailored photoprotection in skin of color (SOC) populations. SOC refers to people with Fitzpatrick skin phototypes (SPTs) III-VI and includes a diverse racial and ethnic background. Though this is a diverse group of people, there are similarities, such as eumelanin-to-phenomelanin ratios and responses to ultraviolet (UV) and visible light (VL) [1]. SOC has higher eumelanin-to-phenomelanin ratios, more mature melanosomes, and enhanced DNA repair capacity compared with lighter skin [2]. The quantity and distribution of melanin in darker skin provide natural photoprotection [2]. DNA damage from UV radiation, usually between 340 and 400 nm, compared with VL, which is 380 to 750 nm, is reduced about threefold in pigmented skin compared with Fitzpatrick Type I-II skin [3,4]. These characteristics of skin in dark populations lead to a misconception that SOC populations are inherently protected from all photodamage and serve as a barrier to proper photoprotection for these populations.

Although the skin cancer incidence is lower in dark skin, mortality is higher, perhaps as a result of late diagnosis or misdiagnosis [4]. Skin cancer in SOC often presents differently than skin cancer in lighter phototypes. Both melanoma and nonmelanoma skin cancer (NMSC) are frequently detected in non-sun-exposed areas. Melanoma can present as acral lentiginous melanoma (ALM) on the palms and soles, subungual melanoma of the nail unit, or mucosal melanoma [2]. Squamous cell carcinoma (SCC) is the most common form of skin cancer in SOC and commonly presents in non-sun-exposed areas, specifically the perianal region, as well as areas of chronic scarring and inflammation [2,5]. UV radiation remains a major risk factor for basal cell carcinomas (BCCs) in SOC; however, these often present as more pigmented, erythematous, or scar-like patches, plaques, or nodules and are therefore often misdiagnosed as melanocytic nevi or seborrheic keratoses [2,5,6].

Although UV damage is not a major risk factor for melanoma and SCC in SOC, these populations are still susceptible to skin damage from UV radiation, resulting in exacerbation of various dermatologic conditions, including pigmentation disorders, photodermatoses, and skin cancer. Post-inflammatory pigmentation disorders can be residual effects of prolonged and repetitive exposure to UVA, increasing the risk for skin cancer. Individuals with SOC are highly susceptible to pigmentary disorders. Post-inflammatory hyperpigmentation (PIH) affects about 15% of people with SOC, while the prevalence of melasma varies geographically, affecting between 30% and 50% of women with SOC [7,8]. Photodermatoses, which are abnormal cutaneous reactions to UVA, may also occur more frequently or with greater severity without proper photoprotection [7]. SOC populations tend to use photoprotection less frequently [2]. This is partly due to limited photoprotection education in SOC populations compared with lighter patient populations [9].

This review aims to comprehensively examine the photoprotection needs for SOC populations, addressing gaps between physiological characteristics and clinical outcomes, evaluating current photoprotection practices, examining educational implications, and considering future directions for photoprotection for SOC. 

## Review

Methods

A literature search was performed using PubMed and Google Scholar. The search was conducted from database inception through July 15, 2026. The following search terms were used in various combinations: "skin of color," "pigmented skin," "dark skin," "Fitzpatrick skin type," "photoprotection," "sunscreen," "photodamage," "photoaging," "ultraviolet radiation," "visible light," "melasma," "post-inflammatory hyperpigmentation," "pigmentary disorders," "photodermatoses," "polymorphous light eruption," "skin cancer," "basal cell carcinoma," "squamous cell carcinoma," "melanoma," "eumelanin," "pheomelanin," and "melanosomes."

Articles were included if they: (1) investigated photoprotection or UV/VL exposure in SOC populations; (2) evaluated sunscreen formulations, photoprotective interventions, photobiology, pigmentary disorders, or sun-protective behaviors; (3) were original research articles, clinical trials, observational studies, systematic reviews, meta-analyses, clinical guidelines, or consensus statements; and (4) were published in English. Studies were excluded if they: (1) did not include SOC populations or relevant subgroup analyses; (2) focused exclusively on noncutaneous photobiology; and (3) consisted solely of conference abstracts or editorials. The literature search, article selection, and synthesis were performed by the authors. As this study was conducted as a narrative review, formal quality assessment and risk-of-bias evaluation were not performed. Evidence was synthesized narratively, with greater emphasis placed on systematic reviews, meta-analyses, clinical guidelines, and higher-quality primary studies when available. A total of 114 articles were included in this narrative review.

Photobiology and mechanisms of photodamage in SOC

UV radiation exerts harmful effects on the skin, particularly in the absence of photoprotection. The two major forms, ultraviolet A (UVA) and ultraviolet B (UVB), differ in skin penetration and biologic impact. UVB is largely absorbed in the epidermis by cellular macromolecules, yet still induces direct DNA damage. In contrast, UVA is less effectively absorbed at the epidermal level, allowing deeper penetration into the dermis, where it promotes oxidative stress rather than direct DNA photoproduct formation [10]. Black epidermis has an SPF of about 13.4, and white epidermis has an SPF of about 3.4 [11]. Although SOC possesses greater baseline photoprotection due to higher constitutive eumelanin content, more mature melanosomes, and enhanced DNA repair capacity, it remains susceptible to both UV-mediated genotoxicity and cytotoxicity [2]. Experimental interventional evidence in SOC (Fitzpatrick III-IV) demonstrates that repetitive suberythemal UVB exposure stimulates melanocyte activation and increases de novo melanin synthesis, offering modest protection against subsequent UV-induced DNA injury. Conversely, UVA-induced tanning results primarily from oxidation and redistribution of pre-existing melanin, providing minimal photoprotection because it does not upregulate melanogenesis [12]. Importantly, repeated exposure to both UVA and UVB generates cumulative cellular and DNA injury, contributing to long-term skin carcinogenesis risk in SOC populations [13].

Beyond UV wavelengths, VL and long-wave UVA1 are significant contributors to pigmentary alteration in SOC. Comparative irradiation studies in darker SOC phenotypes (Fitzpatrick IV-VI) show that VL induces pigmentation that is darker and more persistent than pigmentation induced by UVA1 [14]. Furthermore, VL and UVA1 demonstrate synergistic effects, producing substantially greater pigmentation when combined than when delivered independently [15]. These findings underscore that SOC populations benefit from targeted photoprotection against VL and long-wave UVA1 to reduce the risk of acquired pigmentary disorders, including melasma and PIH.

At the molecular level, UV and VL exposure promote excessive reactive oxygen species (ROS) generation in SOC, partly through catalase upregulation and increased nitric oxide synthesis. When ROS production exceeds endogenous antioxidant defense capacity, oxidative stress ensues, leading to cellular injury, apoptosis, and tissue inflammation [16]. This oxidative burden is clinically relevant, as it can aggravate inflammatory dermatoses in SOC, where dysregulated inflammation is already a key driver of secondary hyperpigmentation.

UV exposure also accelerates structural and functional skin aging. Chronic UVB irradiation induces DNA strand breaks and epidermal accumulation of photoproducts, including cyclobutane pyrimidine dimers and pyrimidine-pyrimidone adducts, which interfere with DNA replication and transcription, ultimately triggering apoptosis [17]. In the dermis, ROS activates cytoplasmic signal transduction pathways in fibroblasts, stimulating matrix metalloproteinase (MMP) activity and promoting collagen and elastin degradation, key processes underlying photoaging [18,19]. Collectively, these biological effects confirm that SOC, while comparatively more protected than lighter skin, is not immune to photodamage and that wavelength-specific, mechanism-based photoprotection is essential for reducing long-term clinical sequelae.

Clinical manifestations of photodamage in SOC

There are numerous clinical manifestations of photodamage in SOC, including pigmentary disorders, photoaging, skin cancer risk, and photodermatoses. Pigmentary disorders, such as melasma and PIH, are the most prevalent forms of dyschromia in SOC populations [20]. These conditions are worsened by UV and VL exposure. Melasma is characterized by brown or gray macules that are more common in sun-exposed areas [21]. People with SOC may present with melasma differently than lighter-skinned individuals. Photoprotection, specifically the regular use of UV and VL broad-spectrum sunscreen, has been found to be efficacious in preventing and treating melasma. Thus, nonadherence to photoprotection in people with SOC can lead to increased exacerbations of this condition [22]. PIH is an acquired hypermelanosis that occurs after injury or inflammation of the skin, more frequently affecting SOC patients, including Hispanics/Latinos, African Americans, Native Americans, Asians, Pacific Islanders, and Middle Easterners. Photoprotection, including sunscreen and protective clothing, is vital for preventing worsening of PIH [7].

Photoaging is another manifestation of light damage that affects people of SOC. People with SOC tend to present with dyspigmentation and solar lentigines with age, as well as “intrinsic” aging features, such as volume loss [23]. Chronic photoexposure in SOC exacerbates aging features, resulting in decreased elasticity at the functional level and loss of the dermal-epidermal junction and organized fibrillar collagen at the histological level [24]. Daily broad-spectrum sun protection with a factor of 30+ is recommended for prevention or reduction of signs of photoaging [25]. Photodermatoses and photodamage are also interconnected in SOC individuals. Photodermatoses are abnormal skin responses to sunlight exposure. Polymorphic light eruption (PMLE) is one of the most common photodermatoses found in SOC and is statistically significantly more common in Black patients compared with White patients (74.3% vs 43.9%, p < 0.001) [26,27]. Hence, patients with photodermatoses require comprehensive photoprotection, as UVB/UVA and VL wavelengths can induce or augment photodermatoses [2].

The relationship between photoprotection and skin cancer remains unclear for SOC individuals. Although skin cancer rates occur at lower rates in SOC populations compared with White individuals, they are associated with greater morbidity and mortality. From 2000 to 2010, melanoma survival improved for White patients, yet SOC continued to have poor survival, with an absolute difference of 25% between Black and White patients [28]. In a study examining incidence rates of skin cancer in England, European age-standardized rates (EASRs) for ALM were highest in the Black ethnic group at 0.85 per 100,000 person-years, compared with the Asian (0.13), Chinese (0.32), Mixed (0.25), Other (0.44), and White (0.36) ethnic groups [29]. Racial disparities in melanoma for SOC are multifactorial and include increased socioeconomic barriers, higher proportions of more aggressive melanoma subtypes, lower screening rates, and diminished skin cancer education for SOC. There are limited studies examining UV exposure and melanoma risk in individuals with SOC, making it difficult to assess the role that photodamage plays in melanoma manifestations in SOC [30]. Similarly, a systematic review found that studies examining the relationship between UV exposure and keratinocyte carcinomas in SOC had mixed results, indicating that further research is needed to assess the role photoprotection may play in skin cancer for SOC [31]. 

Importantly, the role of photoprotection may differ for malignancies arising at special sites. Acral, subungual, and mucosal melanomas, as well as perianal and inflammation-associated SCCs, are largely driven by factors other than UV exposure. As a result, conventional UV-directed photoprotection is unlikely to substantially reduce the risk of malignancies at these sites [2,5,30,31]. Thus, skin cancer prevention in SOC should extend beyond photoprotection alone to include education on skin cancer recognition and awareness of lesions arising in sun-protected and acral locations.

Current photoprotection behaviors and barriers in SOC

SOC populations demonstrated substantially lower sunscreen engagement compared with lighter-skinned populations, with daily use rates of 25% versus 31% among non-Hispanic Whites (NHWs) and Hispanic use ranging from 9.5% to 29.9% compared with 16.5% to 35.9% of Whites [32,33]. Reapplication rates showed even larger disparities (45% SOC vs 76% NHW) [34]. Gender differences were pronounced across all populations studied, with female daily use being 3-12 times higher than male daily use in South Asian and Chinese samples [34]. Economic barriers significantly impacted access, with cost concerns cited by 16% of SOC individuals versus 2% of Whites [32] and lower-income individuals significantly more likely to perceive sunscreen as unaffordable (p = 0.0025) [35]. Product-related barriers, including white cast concerns, affected 25% of SOC individuals versus 7% of Whites, and 41% reported difficulty finding suitable products compared with 22% of Whites [32].

Although lighter skin was valued culturally for aesthetic purposes, sun protection remained low, with motivations focused on preventing tanning and aging rather than cancer prevention [36,37]. Fundamental misconceptions about melanin-based protection contributed to low perceived risk, with nearly half of some populations believing they were not at risk for skin cancer and many attributing protection to “darker skin tone” [38]. Educational gaps were substantial, with only 22% of individuals of color learning about sunscreen from dermatologists compared with 36% of Whites, and most demonstrating limited knowledge about sunscreen types and skin cancer risks [32]. An educational intervention study achieved 94% intended future use, but 54% still cited cost barriers, suggesting education alone cannot overcome economic constraints [38]. These findings indicate that effective interventions must simultaneously address economic barriers, correct risk perception misconceptions, provide culturally adapted products and education, and develop gender-specific messaging. A summary of the photoprotection barriers in SOC is highlighted in Table 1. 

**Table 1:** Summary of photoprotection barriers in skin of color Source: [32-38]

Barrier	Key Findings/Implications
Gender differences	Female sunscreen use was substantially higher than male use across South Asian and Chinese populations.
Economic barriers	Cost concerns were more frequently reported among skin of color (SOC) individuals, and lower-income groups were more likely to perceive sunscreen as unaffordable.
Product barriers	White cast concerns and difficulty finding suitable sunscreen formulations were significantly more common among SOC populations.
Risk perception misconceptions	Many individuals believed darker skin provided sufficient natural protection and perceived themselves at low risk for skin cancer.
Educational gaps	SOC individuals were less likely to receive sunscreen counseling from dermatologists.

Lower participation in sun-protective behaviors in SOC populations is shaped by complex sociocultural factors, educational background, and ingrained societal norms. To address these barriers, culturally relevant education is essential [39]. For instance, a 2025 study found that sun protection behaviors and skin self-examination confidence improved markedly when underserved Spanish- and Punjabi-speaking communities were engaged through in-person, linguistically tailored, community-based programs [40]. These localized successes contrast sharply with the current state of digital health communication. A cross-sectional study of 100 TikTok videos on longitudinal melanonychia found poor educational quality and a notable lack of SOC representation, even in high-visibility videos, underscoring that current digital health communication often fails to meet the needs of diverse populations [41]. To effectively improve health literacy and engagement in SOC communities, messaging should prioritize inclusive visuals and culturally tailored information.

Community engagement and healthcare integration are equally critical for improving photoprotection outcomes. A cluster randomized trial evaluating a Sun Safe Schools intervention found that training and resource support for school leaders significantly increased sun safety practices in elementary schools, illustrating how policy integration with community education and healthcare collaboration can enhance photoprotection in institutional settings [42]. Similarly, community-based interventions implemented through multi-component programs, such as local events and public outreach, have been shown to reduce UV exposure and increase sunscreen use [43]. These results support the effectiveness of programs that broadly engage communities rather than relying solely on clinic-based counseling. However, these programs are not one-size-fits-all. National survey data highlight a drastic divide between rural and urban sun-protection behaviors, likely driven by different occupational and socioeconomic realities [44]. Therefore, the most effective engagement strategies will be those that account for the specific geographic and socioeconomic contexts of the populations they serve.

Dermatology is increasingly moving toward a framework of personalized photoprotection. An international Delphi consensus among dermatologists emphasized tailoring photoprotection strategies according to individual skin cancer risk, phototype, medical history, lifestyle, and treatment goals. Rather than providing generic advice, the panel's 28 recommendations divide care into five distinct domains. These range from clinical management and cancer prevention to the specific needs of darker phototypes. For those with darker skin, the recommendations focus on VL protection and the cosmetic acceptability of the product to ensure long-term adherence [45]. The clinical value of this personalized approach is supported by the PennSCAPE randomized trial, which compared individualized risk communication with standard, generic messaging. The study found that when patients received feedback specifically tailored to their habits and risk factors, they were not just more likely to use sunscreen; they were also more likely to seek shade and perform skin self-examinations [46]. Ultimately, it appears that people sustain these protective behaviors when the guidance feels personally relevant and meaningful to them.

Finally, addressing resource-limited settings remains essential to promoting equitable photoprotection. A 2023 survey study found differences in sunscreen use and affordability perceptions across socioeconomic and ethnic groups, indicating that cost and access barriers vary by income and ethnicity. Lower-income participants and certain racial and ethnic groups were more likely to report sunscreen affordability as a barrier, highlighting the need for public health strategies to reduce financial and logistical obstacles to sun protection [35]. Additionally, an epidemiological study from Brazil identified significant educational inequalities in sun protection practices, demonstrating that lower education levels and socioeconomic status are major barriers to sun protection [47]. Therefore, achieving equitable photoprotection will require policy initiatives that move beyond general awareness to address the structural, financial, and educational inequities that keep underserved populations at risk.

Comprehensive photoprotection strategies

While behavioral adjustments, like seeking shade and avoiding the sun during peak hours, are standard recommendations for reducing UV and VL exposure, environmental inequities often limit how practical these strategies are for individuals with SOC. People with darker skin types face a higher risk of developing pigmentary disorders from prolonged exposure to VL compared with those with lighter skin types, making daily protection essential [9]. For instance, reducing sun exposure between the peak hours of 10 am and 2 pm and seeking out shaded areas are recommended to lower UV and VL exposure [48]. However, neighborhoods with lower socioeconomic status often have noticeably less urban greenspace and natural shade compared with more affluent communities [49]. Because these environmental inequities create a real barrier to basic sun avoidance, clinicians must account for a patient's physical environment when recommending daily photoprotection habits.

Sun-protective clothing offers a highly effective, nonchemical way to defend the skin and eyes, bypassing many of the common barriers associated with sunscreen adherence. Wearing ultraviolet protection factor (UPF)-rated garments, wide-brimmed hats, and UV-filtering sunglasses provides immediate, reliable coverage. For example, using sunglasses reduces the degree of UV radiation that reaches the eye, which may lessen the severity of ocular melanocyte DNA damage [50]. Furthermore, a longitudinal study demonstrated that long-term use of UPF-rated clothing in children lowers cumulative UV exposure and reduces the risk of melanoma by decreasing the number of newly developed pigmented nevi during childhood [51]. For populations with SOC that have historically experienced lower adherence to and education regarding sunscreen use, promoting UPF-rated clothing offers a practical and beneficial alternative. Ultimately, public health campaigns raising awareness about the benefits of sun-protective garments are valuable for all populations, especially individuals with darker skin types.

Achieving consistent daily sunscreen compliance among individuals with SOC requires solving specific cosmetic, financial, and educational challenges. When guiding patients through individualized sunscreen selection, both photoprotection and cosmesis are important factors to consider [9,48]. According to a cross-sectional study by Wang et al., sunscreen adherence is negatively impacted by poor color matching to darker skin tones, visible white casts or residue, and product cost [52]. Furthermore, because coverage against VL limits the number of suitable products available, patients without proper education on key ingredients may struggle to select the most beneficial formulations for their skin type [52]. Understanding these barriers to access and adherence is key to improving compliance and long-term skin health within this patient population.

Cultural shifts and acculturation within diverse communities of color can unexpectedly lead to risky sun behaviors, highlighting the need for culturally tailored clinical counseling. Although artificial tanning is traditionally associated with lighter skin types, recent research highlights rising tanning bed use across broader demographics, which increases cumulative UV exposure and drives up rates of NMSC [53]. For example, a study by Coups et al. found that Hispanic patients with higher levels of acculturation demonstrated increased use of tanning beds and decreased use of sun-protective clothing compared with less-acculturated Hispanic patients [54]. This behavioral difference demonstrates that clinicians cannot rely on assumptions about sun habits based on skin type alone. Instead, ongoing research into behavioral strategies across different cultures is needed to help prevent harmful exposure habits in patients with SOC.

Closing the photoprotection gap in communities of color requires proactive education that spans both dermatology clinic visits and community-based youth programs. Currently, individuals with SOC are less likely to receive photoprotection counseling in the clinical setting, leaving them at greater risk for prolonged UV and VL exposure [9,48]. To deliver safe and thorough care, healthcare providers must educate themselves on evidence-based best practices and actively integrate tailored counseling into routine appointments. Outside the clinic, reaching youth at an early age is a proven way to shape healthy habits before significant cumulative damage occurs. Youth-targeted educational campaigns improve the understanding of sun damage, increase daily sunscreen use, shift attitudes away from the desirability of tanning, and boost adherence to protective strategies early in life [55]. Because this early intervention is paramount for long-term skin health, increasing funding for youth-focused educational seminars in communities of color will be highly beneficial. A summary of these photoprotection strategies is highlighted in Table 2.

**Table 2:** Summary of comprehensive photoprotection strategies Source: [9,48-55]

Photoprotection Strategy	Key Findings/Recommendations
Behavioral Sun Avoidance	Limit sun exposure during peak ultraviolet (UV) hours (10 AM-2 PM) and seek shade when outdoors.
Sun-Protective Clothing	Sunglasses reduce ocular UV exposure and melanocyte DNA damage. Ultraviolet protection factor (UPF)-rated clothing decreases cumulative UV exposure and reduces development of pigmented nevi.
Avoidance of Harmful Behaviors	Tanning bed use increases cumulative UV exposure and skin cancer risk.
Community-Based Education	Youth-targeted sun protection programs increase sunscreen use, reduce tanning desirability, and improve adherence.

Recent developments in photoprotective strategies include oral supplements, such as *Polypodium leucotomos* or oral nicotinamide [56]. There is growing evidence supporting the use of *P. leucotomos* as a photoprotective adjunct [56]. However, larger, more comprehensive trials are needed to further validate these findings. In the field of oral photoprotective strategies, few studies focus primarily on SOC populations. One study highlighted that *P. leucotomos* may lessen persistent darkening following exposure to VL [2]. Further evidence is needed to support the role of *P. leucotomos* in pigmentary conditions affecting SOC, such as melasma and PIH [2,57,58]. The evidence supporting the utility of oral nicotinamide as an adjunctive photoprotective strategy, particularly in people with SOC, is also limited [2]. One study demonstrated that oral tranexamic acid significantly reduced Melasma Area and Severity Index (MASI) scores compared with topical or intradermal formulations, supporting its use as a photoprotective adjunct in randomized controlled trials [59]. A meta-analysis demonstrated that oral tranexamic acid showed high efficacy for the treatment of melasma and received high scores in terms of patient satisfaction [60]. Although the number of studies focused exclusively on SOC cohorts evaluating tranexamic acid is limited, one analysis demonstrated that intradermal tranexamic acid significantly reduced MASI scores with a limited side-effect profile in individuals with Fitzpatrick skin types IV-V [61]. With this evidence in mind, it is important to emphasize that oral photoprotective strategies should be considered adjuncts and should not replace the use of sunscreens and other photoprotective methods discussed in this review.

Sunscreen formulation considerations for SOC

Sunscreen use should be tailored to SPT. Individuals with SOC are more prone to developing hyperpigmentation disorders [62]. Therefore, for people with SOC (Fitzpatrick skin types IV-VI), the priority is defending against UVA rays and VL, which are primary triggers for hyperpigmentation [13]. Studies have shown that VL, once considered harmless, is, in fact, a major driver of long-lasting pigmentation in SOC. Furthermore, studies overall show that VL induces more intense and persistent pigmentation than UVA [7,63].

For individuals with SOC, daily use of SPF 30+ sunscreens with a UVA-PF/SPF ratio of <1.5 is recommended [9,13]. In contrast, lighter phototypes (I-III) are more prone to erythema and sunburn, necessitating higher SPF levels (50+) and a recommended UVA-PF/SPF ratio of <3. Importantly, UVA, VL, and infrared A are all known to penetrate deeply into the skin and contribute to photoaging, so protection against all three can be beneficial [13].

Most standard sunscreens block only UVA and UVB. However, VL protection generally requires an opaque barrier [64]. For this reason, dermatologists recommend tinted sunscreens containing iron oxides, which protect against VL while providing coverage that minimizes visible discoloration [13,63]. This is especially beneficial for the prevention and management of hyperpigmentation disorders.

Despite these benefits, individuals with SOC are less likely to regularly use sunscreen, partly due to inadequate education about photodamage and partly due to poor cosmetic acceptability. Many sunscreens leave a white residue or “cast,” discouraging use [65]. Developing formulations that avoid white residue and better match diverse skin tones is crucial for improving satisfaction and adherence [2]. However, clinical assessments of “universal” tinted sunscreens show that many still fail to provide an adequate color match for the darkest or fairest complexions, highlighting the need for expanded shade diversity in sunscreen formulations [66]. Unfortunately, pharmacological testing of SPF and UVA-PF sunscreens is based on erythema-based methodology, which undermines the utility of selected formulations in individuals with Fitzpatrick skin types V-VI [67]. Instead, using endpoints such as persistent pigment darkening may be a more suitable and inclusive approach for evaluating the efficacy of photoprotection in SOC populations [68]. Additionally, there is no standardized ISO method for VL-PF assessment at this time, which further limits current understanding in the field [69].

Recent advances in sunscreen formulation have focused on incorporating antioxidants to provide broader protection and address the specific needs of SOC. UV and VL exposure can generate ROS and reactive nitrogen species (RNS), which trigger inflammation and PIH, the latter of which tends to be more persistent in darker skin. This pigmentation is due to the activation of Opsin-3 (OPN3) receptors by VL. Unlike the transient pathways activated by UV, OPN3 induces the formation of a stable, multimeric tyrosinase/dopachrome tautomerase (DCT) complex. This results in sustained melanin production and long-lasting clinical hyperpigmentation in SOC [13,70,71]. Antioxidants such as vitamin E, vitamin C, ferulic acid, niacinamide, and ectoine neutralize these species and mitigate DNA damage in ways that traditional UV filters cannot. Specifically, niacinamide has been shown to support DNA repair, suppress inflammatory mediators, and activate cellular antioxidant pathways, further reinforcing its role as an effective adjunct in photoprotective formulations [72]. Industry adoption is already widespread; a 2023 review noted that over 95% of commercial sunscreens now include at least one antioxidant, most commonly vitamins E and C [47].

In addition to UV filters and antioxidants, emerging evidence supports the inclusion of depigmenting agents directly within sunscreen formulations to address pigmentary concerns, particularly in SOC. A randomized, investigator-blinded clinical trial demonstrated that a broad-spectrum sunscreen containing niacinamide and sclareolide significantly reduced pigmentation and erythema associated with PIH compared with unprotected skin. Specifically, ΔITA° (a validated inverse correlate of skin pigmentation) improved by nearly 78% in protected, non-stripped areas and by 49% in inflamed, stripped areas [73]. Similarly, another clinical study found that a multifunctional facial sunscreen with 2% niacinamide improved overall pigmentation and skin tone uniformity over 28 and 56 days, supporting the use of niacinamide-containing sunscreens for pigmentary improvement in SOC [74]. Together, these findings highlight a shift toward the production of multifunctional, cosmetically refined products that treat existing pigmentary issues while simultaneously preventing new damage in SOC populations.

Special populations and conditions

Pediatric Considerations in SOC

Children with SOC demonstrate lower‑frequency sunscreen use and encounter cultural, socioeconomic, and product‑access barriers, as documented in fifth‑grade surveys and market analyses of sunscreen availability [75-77]. Although higher eumelanin levels afford some UV attenuation, they do not shield against UVA‑1 and VL wavelengths that precipitate hyperpigmentation in pediatric patients [63,78]. Sunburn history is a well-established risk factor for cutaneous melanoma. Epidemiologic evidence demonstrates a dose-response relationship, with increasing numbers of sunburns across childhood, adolescence, and adulthood associated with progressively higher melanoma risk [79,80]. Therefore, early introduction of broad‑spectrum, tinted sunscreens containing iron‑oxide pigments is essential; these formulations block UV and VL while providing a cosmetically acceptable finish that improves adherence in diverse skin tones [63,76,81]. Consistent use of such products can reduce pigmentary sequelae, support healthy skin‑tone development, and help establish lifelong sun‑safe habits in children with SOC [76].

Melasma Management and Prevention

Clinical trials demonstrate that melasma relapse rates fall dramatically when sunscreens are formulated to block both UV radiation and short‑wavelength VL; a double‑blind randomized study showed superior outcomes with a broad‑spectrum sunscreen that incorporated iron‑oxide pigments, which attenuate VL‑induced pigmentation while maintaining high SPF protection [82]. Systematic reviews of randomized controlled trials further confirm that adding a VL‑protective component to sunscreen enhances the efficacy of hydroquinone‑based regimens, reducing melasma severity scores more than UV‑only protection [83]. The international Delphi consensus explicitly identifies broad‑spectrum, tinted sunscreens containing iron oxide as a cornerstone of melasma therapy for patients of color, emphasizing that VL is a potent trigger of repigmentation in darker skin types and that cosmetic acceptability drives adherence [81]. Real‑world evaluations of iron‑oxide‑enriched formulations report that 4.5% yellow iron oxide effectively blocks VL‑induced melanin synthesis, offering a practical strategy to prevent pigmentary relapse while avoiding the white‑cast associated with traditional sunscreens [63,84].

Photoprotection in Inflammatory Dermatoses

For patients with SOC who have inflammatory dermatoses, effective photoprotection requires targeted defense against long-wavelength UVA (UVA-1) and VL (400 to 700 nm), both of which drive lasting pigmentation and inflammation [13,85]. Unlike UVB rays, UVA-1 and VL penetrate deep into the dermis. This deeper reach activates inflammatory cascades in acne and rosacea and worsens PIH through sustained melanin production [13,85]. Traditional UV-only sunscreens fail to prevent this reaction. However, clinical data show that broad-spectrum sunscreens with a UVA-PF of at least two-thirds, paired with VL filters such as iron oxides or organic absorbers, significantly reduce porphyrin production and decrease inflammatory acne lesions, although reductions in PIH severity in those specific trials did not reach statistical significance [86,87].

Adding microfine iron oxide pigments solves two problems at once: it physically blocks short-wavelength VL and adds a tint that eliminates the chalky white cast that often discourages daily use on darker skin [63,88]. In fact, randomized evaluations of 33 sunscreen products confirm that iron oxide-enriched formulations offer significantly better pigmentation control and VL protection than conventional untinted sunscreens [89,90]. These results can be improved even further by adding topical anti-inflammatory agents to the formulation. For example, a prospective study showed that sunscreens combining broad-spectrum filters with sclareolide and niacinamide successfully prevent PIH after combined UV and VL exposure [73]. Because of this, standard clinical care for patients with acne, rosacea, and eczema in SOC should prioritize high-UVA-PF, iron oxide-tinted formulations that suppress light-induced inflammation, prevent PIH, and blend well enough to encourage consistent use.

HIV‑Associated Dermatoses

Immunosuppression in people living with HIV (PLWH) amplifies photosensitivity and predisposes individuals to pigmentary disorders such as hyperpigmentation and post-inflammatory dyschromia, especially in those with darker skin tones [91,92]. Although direct trials of photoprotection in SOC cohorts of PLWH are scarce, epidemiologic work in patients from Thailand showed that 16% of PLWH reported photosensitivity and that only about one-third used sunscreen daily, underscoring a gap in preventive care [93]. Broad-spectrum sunscreens that deliver a UVA-PF of at least two-thirds and contain iron oxide pigments provide dual UV/VL protection while offering a tint that matches melanin-rich skin, eliminating the “white cast” that often deters adherence in SOC [62]. Clinicians should therefore prescribe tinted, high-UVA/VL formulations as a core component of routine dermatologic management for PLWH while vigilantly monitoring for opportunistic skin infections and pigmentary flares that may arise from UV/VL exposure.

Photoprotection in Vitiligo, Lichen Planus Pigmentosus (LPP), and Erythema Dyschromicum Perstans (EDP)

Vitiligo, LPP, and EDP represent three pigmentary disorders with distinct photobiological considerations that are often overlooked in photoprotection discussions. In vitiligo, depigmented patches lack functional melanocytes and therefore have no endogenous UV defense, placing affected skin at heightened risk for sunburn and cumulative photodamage despite the overall lower melanoma incidence observed in vitiligo cohorts [94]. Photoprotection in vitiligo must therefore balance the need to protect depigmented areas with the recognized role of controlled UVB exposure (narrowband UVB phototherapy) as a mainstay of repigmentation treatment [95]. Broad-spectrum sunscreens with an iron oxide tint are recommended for depigmented areas during daily activities to prevent acute UV injury while preserving the option for supervised therapeutic UV exposure.

LPP is a chronic T-cell-mediated interface dermatitis that preferentially affects sun-exposed areas in individuals with darker SPTs, manifesting as slate-gray to brownish-black macules [96]. UV and VL exposure have been implicated as triggers, and strict photoprotection is consistently recommended as a first-line management strategy alongside topical calcineurin inhibitors and systemic agents [97,98]. Because LPP involves dermal melanin deposition driven in part by UV- and VL-mediated inflammation, broad-spectrum photoprotection with tinted formulations that block both UVA and visible wavelengths may help prevent disease exacerbation and progression [98].

EDP (ashy dermatosis) is a related acquired dermal hypermelanosis of uncertain etiology that predominantly affects Latin American and South Asian populations, presenting as asymptomatic, slowly progressive ash-gray macules [99]. Although its exact pathogenesis remains debated, UV radiation and contact allergens have been suggested as contributing factors, and photoprotection is regarded as a foundational element of management [99,100]. Given that both LPP and EDP involve deep dermal pigment that is resistant to topical depigmenting agents, preventing further UV-driven melanin deposition through comprehensive photoprotection, including VL filtration via iron oxide sunscreens, is particularly important [100].

Vitamin D Considerations in Photoprotection for SOC

Cutaneous vitamin D synthesis depends on UVB exposure, and higher epidermal melanin levels reduce UVB penetration, placing darker-skinned individuals at greater risk for vitamin D insufficiency [101]. This creates a clinical tension in photoprotection counseling: concerns have been raised that rigorous sun avoidance and sunscreen use could further suppress vitamin D production in populations already at risk of deficiency [4]. However, real-world evidence suggests that sunscreen use has a minimal impact on vitamin D status, largely because products are applied infrequently and at subtherapeutic thickness [102].

Clothing has been shown to markedly inhibit cutaneous vitamin D synthesis, whereas sunscreen under typical use conditions may have a smaller effect [103]. For darker-skinned individuals living at higher latitudes or maintaining predominantly indoor lifestyles, lower thresholds for screening serum 25-hydroxyvitamin D have been suggested, given the increased risk of deficiency related to reduced UVB-mediated synthesis [104]. Despite these recommendations, formal evidence-based guidelines for screening frequency and supplementation strategies remain lacking for SOC populations, representing a critical gap in the literature [105].

Evidence gaps and research needs

All sources addressing research representation documented that SOC populations have been historically underinformed about photoprotection compared with lighter-skinned individuals, with current messaging primarily addressing lighter skin types and the importance of photoprotection in these populations being understudied and underestimated [9,62,106]. Most critically, the literature identified a complete absence of studies examining whether photoprotection behaviors reduce skin cancer mortality in SOC populations, despite the paradox that these groups have lower incidence but significantly higher mortality rates, with five-year melanoma survival of only 72.2% for African Americans compared with 89.6% for Caucasians [106,107]. Regarding optimal protection levels by phototype, no source provided varying SPF recommendations, only universal guidance for SPF 50+ and broad-spectrum UVA/UVB protection [62]. Educational resources should therefore emphasize VL protection through tinted sunscreens containing iron oxide for darker skin types, given the disproportionate burden of hyperpigmentation disorders in SOC populations [9].

Future directions and innovations

Advancements in sunscreen technology aim to improve the efficacy, cosmetic acceptability, and feasibility of photoprotection in SOC populations. Tinted sunscreens have emerged as a validated innovation that minimizes the white cast associated with traditional formulations while improving patient adherence and maintaining high UV coverage [108]. More recent developments demonstrate that tinted sunscreens also attenuate VL-induced photodamage, a critical addition given the susceptibility of SOC to pigmentary injury [109]. In parallel, new filter technologies have expanded long-wavelength protection, including the incorporation of methoxypropylamino cyclohexenylidene ethoxyethylcyanoacetate, a selective UVA1 blocker now integrated into advanced sunscreen formulations [110]. Additionally, infrared protection appears most effective when sunscreen has adequate dermal penetration, although this may require longer application times, which has implications for real-world adherence [111].

Novel synthetic UV filters further strengthen broad-spectrum photoprotection. Agents such as bemotrizinol and bisoctrizole provide extensive UVA and UVB coverage; however, they are not yet approved in the United States for over-the-counter use. Continued innovation in filter chemistry and formulation science represents a meaningful step toward reducing UV-associated skin neoplasms [112]. Beyond synthetic filters, naturally derived photoprotective compounds from plants and marine organisms have gained increasing attention. Flavonoids, abundant in plants, exhibit dual photoprotective potential by scavenging ROS and absorbing UV radiation, positioning them as promising candidates for adjunctive sunscreen protection [113]. Similarly, marine organisms produce mycosporine-like amino acids (MAAs), which demonstrate strong UV-absorbing capacity and represent another potential bio-inspired sunscreen additive [114]. While these compounds show mechanistic potential, further research is required to evaluate their stability, bioavailability, and clinical effectiveness in sunscreen formulations, particularly in SOC populations.

A limitation of this review is that the evidence base is predominantly North American, reflecting a paucity of studies from other regions; future research should prioritize expanding the evidence base to include more geographically diverse populations. Furthermore, as a narrative review, this study was intended to provide a broad synthesis of the available literature rather than a systematic evaluation of all eligible studies.

## Conclusions

Comprehensive photoprotection is essential for SOC populations. SOC individuals face unique manifestations of photodamage. Effective photoprotection in SOC requires extension beyond traditional UVB protection to meet the specific needs of SOC. Additionally, targeted education, community engagement, and barriers to care need to be addressed to improve outcomes. It is important for the dermatology community to commit to inclusivity and advocate for equitable access to photoprotection resources for all. 

## References

[1] Taylor SC (2002). Skin of color: biology, structure, function, and implications for dermatologic disease. J Am Acad Dermatol.

[2] Krutmann J, Piquero-Casals J, Morgado-Carrasco D, Granger C, Trullàs C, Passeron T, Lim HW (2023). Photoprotection for people with skin of colour: needs and strategies. Br J Dermatol.

[3] Al-Sadek T, Yusuf N (2024). Ultraviolet radiation biological and medical implications. Curr Issues Mol Biol.

[4] Fajuyigbe D, Young AR (2016). The impact of skin colour on human photobiological responses. Pigment Cell Melanoma Res.

[5] Gloster HM Jr, Neal K (2006). Skin cancer in skin of color. J Am Acad Dermatol.

[6] Karampinis E, Georgopoulou KE, Kampra E (2024). Clinical and dermoscopic patterns of basal cell carcinoma and its mimickers in skin of color: a practical summary. Medicina (Kaunas).

[7] Davis EC, Callender VD (2010). Postinflammatory hyperpigmentation: a review of the epidemiology, clinical features, and treatment options in skin of color. J Clin Aesthet Dermatol.

[8] Morgado-Carrasco D, Piquero-Casals J, Granger C, Trullàs C, Passeron T (2022). Melasma: the need for tailored photoprotection to improve clinical outcomes. Photodermatol Photoimmunol Photomed.

[9] Seck S, Hamad J, Schalka S, Lim HW (2023). Photoprotection in skin of color. Photochem Photobiol Sci.

[10] Holick MF (2016). Biological effects of sunlight, ultraviolet radiation, visible light, infrared radiation and vitamin D for health. Anticancer Res.

[11] Tsai J, Chien AL (2023). Reinforcing photoprotection for skin of color: a narrative review. Dermatol Ther (Heidelb).

[12] Miyamura Y, Coelho SG, Schlenz K (2011). The deceptive nature of UVA tanning versus the modest protective effects of UVB tanning on human skin. Pigment Cell Melanoma Res.

[13] Passeron T, Lim HW, Goh CL (2021). Photoprotection according to skin phototype and dermatoses: practical recommendations from an expert panel. J Eur Acad Dermatol Venereol.

[14] Mahmoud BH, Ruvolo E, Hexsel CL (2010). Impact of long-wavelength UVA and visible light on melanocompetent skin. J Invest Dermatol.

[15] Kohli I, Chaowattanapanit S, Mohammad TF (2018). Synergistic effects of long-wavelength ultraviolet A1 and visible light on pigmentation and erythema. Br J Dermatol.

[16] de Jager TL, Cockrell AE, Du Plessis SS (2017). Ultraviolet light induced generation of reactive oxygen species. Adv Exp Med Biol.

[17] Vechtomova YL, Telegina TA, Buglak AA, Kritsky MS (2021). UV radiation in DNA damage and repair involving DNA-photolyases and cryptochromes. Biomedicines.

[18] Scharffetter-Kochanek K, Brenneisen P, Wenk J (2000). Photoaging of the skin from phenotype to mechanisms. Exp Gerontol.

[19] Luze H, Nischwitz SP, Zalaudek I, Müllegger R, Kamolz LP (2020). DNA repair enzymes in sunscreens and their impact on photoageing - a systematic review. Photodermatol Photoimmunol Photomed.

[20] Huerth KA, Hassan S, Callender VD (2019). Therapeutic insights in melasma and hyperpigmentation management. J Drugs Dermatol.

[21] Fatima S, Braunberger T, Mohammad TF, Kohli I, Hamzavi IH (2020). The role of sunscreen in melasma and postinflammatory hyperpigmentation. Indian J Dermatol.

[22] Desai SR, Alexis AF, Elbuluk N, Grimes PE, Weiss J, Hamzavi IH, Taylor SC (2024). Best practices in the treatment of melasma with a focus on patients with skin of color. J Am Acad Dermatol.

[23] Kaltchenko MV, Chien AL (2025). Photoaging: current concepts on molecular mechanisms, prevention, and treatment. Am J Clin Dermatol.

[24] Langton AK, Alessi S, Hann M, Chien AL, Kang S, Griffiths CE, Watson RE (2019). Aging in skin of color: disruption to elastic fiber organization is detrimental to skin's biomechanical function. J Invest Dermatol.

[25] Venkatesh S, Maymone MB, Vashi NA (2019). Aging in skin of color. Clin Dermatol.

[26] Wadhwani AR, Sharma VK, Ramam M, Khaitan BK (2013). A clinical study of the spectrum of photodermatoses in dark-skinned populations. Clin Exp Dermatol.

[27] Hamel R, Mohammad TF, Chahine A (2020). Comparison of racial distribution of photodermatoses in USA academic dermatology clinics: a multicenter retrospective analysis of 1080 patients over a 10-year period. Photodermatol Photoimmunol Photomed.

[28] Brunsgaard EK, Wu YP, Grossman D (2023). Melanoma in skin of color: part I. epidemiology and clinical presentation. J Am Acad Dermatol.

[29] Ahmed S, van Bodegraven B, Proby C (2025). Ethnicity and the epidemiology of skin cancer incidence: a national retrospective population-based study in England, 2013-20. Br J Dermatol.

[30] Brunsgaard EK, Jensen J, Grossman D (2023). Melanoma in skin of color: part II. racial disparities, role of UV, and interventions for earlier detection. J Am Acad Dermatol.

[31] Kolitz E, Lopes FCPS, Arffa M, Pineider J, Bogucka R, Adamson AS (2022). UV exposure and the risk of keratinocyte carcinoma in skin of color: a systematic review. JAMA Dermatol.

[32] Wang JY, Patel P, Philip R (2024). Sunscreen practices and preferences of skin of color patients. J Drugs Dermatol.

[33] Weiss J, Kirsner RS, Hu S (2012). Trends in primary skin cancer prevention among US Hispanics: a systematic review. J Drugs Dermatol.

[34] Toor H, Gill MS, Ahad T (2025). Beliefs and attitudes towards sunscreen and sun protection practices among South Asians in British Columbia: a survey study. Photodermatol Photoimmunol Photomed.

[35] Ullman LE, Nasir-Moin M, Hoffman V, Ghadersohi S, Swartzman I, de Weever M, Augustin M (2024). Sunscreen use and affordability attitudes based on ethnicity, socioeconomic status, and Fitzpatrick skin type. Arch Dermatol Res.

[36] Yan S, Xu F, Yang C (2015). Demographic differences in sun protection beliefs and behavior: a community-based study in Shanghai, China. Int J Environ Res Public Health.

[37] Buchanan Lunsford N, Berktold J, Holman DM, Stein K, Prempeh A, Yerkes A (2018). Skin cancer knowledge, awareness, beliefs and preventive behaviors among black and hispanic men and women. Prev Med Rep.

[38] Chapman LW, Ochoa A, Tenconi F, Herman A (2015). Dermatologic health literacy in underserved communities: a case report of South Los Angeles middle schools. Dermatol Online J.

[39] King A, Sabbah SG, Bose R, Heath CR, Silverberg NB (2025). Photoprotection in skin of color: a scoping review of barriers, behaviors, and pediatric considerations. Pediatr Dermatol.

[40] Sanchez-Anguiano ME, Zarate Jarquin JC, Ledesma Y, Maverakis E, Ashbaugh Ortega A (2025). From knowledge gaps to confidence: a pilot study evaluating a multilingual skin cancer educational intervention in underserved Spanish- and Punjabi-speaking communities. Int J Womens Dermatol.

[41] Albucker SJ, Lipner SR (2023). Social media creators are far from nailing it: a cross-sectional analysis of 100 longitudinal melanonychia TikTok videos shows poor educational content and lack of skin of color representation. J Cutan Med Surg.

[42] Reynolds KD, Buller DB, Buller MK, Massie K, Berteletti J, Ashley J, Meenan R (2020). Randomized controlled trial evaluating an intervention supporting implementation of sun safety policies in California public elementary schools. Prev Med.

[43] Sandhu PK, Elder R, Patel M (2016). Community-wide interventions to prevent skin cancer: two community guide systematic reviews. Am J Prev Med.

[44] Dona AC, Jewett PI, Henning-Smith C, Ahmed RL, Wei ML, Lazovich D, Vogel RI (2024). Rural-urban differences in sun exposure and protection behaviors in the United States. Cancer Epidemiol Biomarkers Prev.

[45] de Troya-Martín M, Rodríguez-Martínez A, Rivas-Ruiz F (2025). Personalized photoprotection: expert consensus and recommendations from a Delphi study among dermatologists. Photodermatol Photoimmunol Photomed.

[46] Glanz K, Volpicelli K, Jepson C, Ming ME, Schuchter LM, Armstrong K (2015). Effects of tailored risk communications for skin cancer prevention and detection: the PennSCAPE randomized trial. Cancer Epidemiol Biomarkers Prev.

[47] de Menezes-Júnior LA (2025). Educational inequalities in sun protection practices among Brazilian adults. Sci Rep.

[48] Rigel DS, Taylor SC, Lim HW (2022). Photoprotection for skin of all color: consensus and clinical guidance from an expert panel. J Am Acad Dermatol.

[49] Rahman MS, Meenar M, Labib SM, Howell T, Adlakha D, Woodward B (2024). Unveiling environmental justice in two US cities through greenspace accessibility and visible greenness exposure. Urban For Urban Green.

[50] Dhomen N, Mundra PA, Marais R (2021). Sunglasses to hide behind may also prevent melanoma of the eyes. Br J Cancer.

[51] Harrison SL, Buettner PG, Nowak MJ (2023). Sun-protective clothing worn regularly during early childhood reduces the number of new melanocytic nevi: the North Queensland sun-safe clothing cluster randomized controlled trial. Cancers (Basel).

[52] Wang JY, Patel P, Philip R (2024). Sunscreen practices and preferences of skin of color patients. J Drugs Dermatol.

[53] Watson M, Holman DM, Maguire-Eisen M (2016). Ultraviolet radiation exposure and its impact on skin cancer risk. Semin Oncol Nurs.

[54] Coups EJ, Stapleton JL, Hudson SV, Medina-Forrester A, Natale-Pereira A, Goydos JS (2012). Sun protection and exposure behaviors among Hispanic adults in the United States: differences according to acculturation and among Hispanic subgroups. BMC Public Health.

[55] Reyes-Marcelino G, Wang R, Gultekin S (2021). School-based interventions to improve sun-safe knowledge, attitudes and behaviors in childhood and adolescence: a systematic review. Prev Med.

[56] Natarelli N, Aflatooni S, Stankiewicz K, Correa-Selm L, Sivamani RK (2025). Oral supplements and photoprotection: a systematic review. J Med Food.

[57] Segars K, McCarver V, Miller RA (2021). Dermatologic applications of Polypodium leucotomos: a literature review. J Clin Aesthet Dermatol.

[58] Nestor M, Bucay V, Callender V, Cohen JL, Sadick N, Waldorf H (2014). Polypodium leucotomos as an adjunct treatment of pigmentary disorders. J Clin Aesthet Dermatol.

[59] Calacattawi R, Alshahrani M, Aleid M (2024). Tranexamic acid as a therapeutic option for melasma management: meta-analysis and systematic review of randomized controlled trials. J Dermatolog Treat.

[60] Chen LY, Kang YN, Hoang KD, Chen KH, Chen C (2025). Intradermal injection of tranexamic acid for the treatment of adult melasma: a systematic review and meta-analysis of randomized controlled trials. Facial Plast Surg Aesthet Med.

[61] Hosfiar VA, Sitohang IB, Rahmayunita G (2025). Effectiveness and safety of intradermal tranexamic acid injection as an adjunctive treatment for melasma in skin type IV-V: a double-blind randomized controlled trial. J Clin Aesthet Dermatol.

[62] Taylor SC, Alexis AF, Armstrong AW, Chiesa Fuxench ZC, Lim HW (2022). Misconceptions of photoprotection in skin of color. J Am Acad Dermatol.

[63] Zhou C, Lee C, Salas J, Luke J (2024). Guide to tinted sunscreens in skin of color. Int J Dermatol.

[64] Guan LL, Lim HW, Mohammad TF (2021). Sunscreens and photoaging: a review of current literature. Am J Clin Dermatol.

[65] Song H, Beckles A, Salian P, Porter ML (2020). Sunscreen recommendations for patients with skin of color in the popular press and in the dermatology clinic. Int J Womens Dermatol.

[66] Bardhi R, Mokhtari M, Masood M (2024). Subjective and objective assessment of color match of universal tinted sunscreens in Fitzpatrick skin phototypes I-VI. Photodermatol Photoimmunol Photomed.

[67] Calderon Alonso AI, Lim HW, Tsao H (2026). Sunscreen and skin cancer prevention: photobiology, clinical evidence, and emerging controversies. Arch Dermatol Res.

[68] Moyal D, Chardon A, Kollias N (2000). Determination of UVA protection factors using the persistent pigment darkening (PPD) as the end point (Part 1). Calibration of the method. Photodermatol Photoimmunol Photomed.

[69] Lim HW, Kohli I, Granger C (2021). Photoprotection of the skin from visible light-induced pigmentation: current testing methods and proposed harmonization. J Invest Dermatol.

[70] Boo YC (2021). Mechanistic basis and clinical evidence for the applications of nicotinamide (niacinamide) to control skin aging and pigmentation. Antioxidants.

[71] Setty SR (2018). Opsin3-A link to visible light-induced skin pigmentation. J Invest Dermatol.

[72] Jesus A, Mota S, Torres A (2023). Antioxidants in sunscreens: which and what for?. Antioxidants (Basel).

[73] Passeron T, Brown A, Furmanczyk M, Foyaca M, Trullas C, Piquero‑Casals J (2025). An investigator‑blinded, randomized trial of a broad‑spectrum sunscreen containing sclareolide and niacinamide for the prevention of post‑inflammatory hyperpigmentation in skin of color. Dermatol Ther (Heidelb).

[74] Sampaio S, Rennó I, Peyrot A (2025). Improvement in pigmentation and skin tone uniformity after 28 and 56 days of use of a multifunctional facial sunscreen with 2% niacinamide: clinical outcomes and in vitro permeation results. J Am Acad Dermatol.

[75] Correnti CM, Klein DJ, Elliott MN (2018). Racial disparities in fifth-grade sun protection: evidence from the Healthy Passages study. Pediatr Dermatol.

[76] Cole Y, Ilyas AM, Ilyas EN (2023). Availability of adequate photoprotection for skin of color. Cureus.

[77] Cestari T, Buster K (2017). Photoprotection in specific populations: children and people of color. J Am Acad Dermatol.

[78] Mitchell KN, Tay YK, Heath CR, Silverberg NB (2021). Review article: emerging issues in pediatric skin of color, part 2. Pediatr Dermatol.

[79] Kwa M, Ravi M, Elhage K, Schultz L, Lim HW (2025). The risk of ultraviolet exposure for melanoma in Fitzpatrick skin types I-IV: a 20-year systematic review with meta-analysis for sunburns. J Eur Acad Dermatol Venereol.

[80] Dennis LK, Vanbeek MJ, Beane Freeman LE, Smith BJ, Dawson DV, Coughlin JA (2008). Sunburns and risk of cutaneous melanoma: does age matter? A comprehensive meta-analysis. Ann Epidemiol.

[81] Sarkar R, Desai SR, Sinha S (2025). Delphi consensus on melasma management by international experts and pigmentary disorders society. J Eur Acad Dermatol Venereol.

[82] Castanedo-Cazares JP, Hernandez-Blanco D, Carlos-Ortega B, Fuentes-Ahumada C, Torres-Álvarez B (2014). Near-visible light and UV photoprotection in the treatment of melasma: a double-blind randomized trial. Photodermatol Photoimmunol Photomed.

[83] Pennitz A, Kinberger M, Avila Valle G, Passeron T, Nast A, Werner RN (2022). Self-applied topical interventions for melasma: a systematic review and meta-analysis of data from randomized, investigator-blinded clinical trials. Br J Dermatol.

[84] Ruvolo E, Fair M, Hutson A, Liebel F (2018). Photoprotection against visible light-induced pigmentation. Int J Cosmet Sci.

[85] Piquero-Casals J, Morgado-Carrasco D, Rozas-Muñoz E (2023). Sun exposure, a relevant exposome factor in acne patients and how photoprotection can improve outcomes. J Cosmet Dermatol.

[86] Puaratanaarunkon T, Asawanonda P (2022). A randomized, double blinded, split-face study of the efficacy of using a broad spectrum sunscreen with anti-inflammatory agent to reduce post inflammatory hyperpigmentation after picosecond laser. Clin Cosmet Investig Dermatol.

[87] Shenoy A, Madan R (2020). Post-inflammatory hyperpigmentation: a review of treatment strategies. J Drugs Dermatol.

[88] Martini APM, Maia Campos PMBG (2018). Influence of visible light on cutaneous hyperchromias: clinical efficacy of broad-spectrum sunscreens. Photodermatol Photoimmunol Photomed.

[89] Schalka S, de Paula Corrêa M, Sawada LY, Canale CC, de Andrade TN (2019). A novel method for evaluating sun visible light protection factor and pigmentation protection factor of sunscreens. Clin Cosmet Investig Dermatol.

[90] Dumbuya H, Grimes PE, Lynch S (2020). Impact of iron-oxide containing formulations against visible light-induced skin pigmentation in skin of color individuals. J Drugs Dermatol.

[91] Mohseni Afshar Z, Goodarzi A, Emadi SN (2023). A comprehensive review on HIV-associated dermatologic manifestations: from epidemiology to clinical management. Int J Microbiol.

[92] Ameen M (2013). The impact of human immunodeficiency virus-related diseases on pigmented skin types. Br J Dermatol.

[93] Chaiyabutr C, Sripanidkulchai K, Silpa-Archa N (2025). Prevalence of self reported photosensitivity and sunscreen use among people living with HIV in Thailand. Sci Rep.

[94] Taïeb A, Ezzedine K (2013). Vitiligo: the white armour?. Pigment Cell Melanoma Res.

[95] Pan JY, Sarkany RPR (2011). A comparison of NB-UVB and PUVA in the treatment of vitiligo. Vitiligo - Management and Therapy.

[96] Kumarasinghe SPW, Pandya A, Chandran V (2019). A global consensus statement on ashy dermatosis, erythema dyschromicum perstans, lichen planus pigmentosus, idiopathic eruptive macular pigmentation, and Riehl's melanosis. Int J Dermatol.

[97] Tejaswaroop M, Karishni C (2026). Updates in the management of lichen planus pigmentosus: a narrative review. Pigment Int.

[98] Khanna N, Rasool S (2011). Facial melanoses: Indian perspective. Indian J Dermatol Venereol Leprol.

[99] Cestari TF, Dantas LP, Boza JC (2014). Acquired hyperpigmentations. An Bras Dermatol.

[100] Passeron T, Desai SR, Abdallah M (2026). Global consensus on the management of melanin hyperpigmentation disorders. J Eur Acad Dermatol Venereol.

[101] Xiang F, Lucas R, de Gruijl F, Norval M (2015). A systematic review of the influence of skin pigmentation on changes in the concentrations of vitamin D and 25-hydroxyvitamin D in plasma/serum following experimental UV irradiation. Photochem Photobiol Sci.

[102] Passeron T, Bouillon R, Callender V (2019). Sunscreen photoprotection and vitamin D status. Br J Dermatol.

[103] Datta P, Philipsen PA, Idorn LW, Wulf HC (2021). Low vitamin D in dark-skinned immigrants is mainly due to clothing habits and low UVR exposure: a Danish observational study. Photochem Photobiol Sci.

[104] Webb AR, Kazantzidis A, Kift RC, Farrar MD, Wilkinson J, Rhodes LE (2018). Colour counts: Sunlight and skin type as drivers of vitamin D deficiency at UK latitudes. Nutrients.

[105] Abdul-Jabbar S, Nebechi C, McClelland GR (2025). Ethnic differences in response to oral vitamin D supplementation: a systematic review and meta-analysis. Nutr Rev.

[106] Tsai J, Chien AL (2022). Photoprotection for skin of color. Am J Clin Dermatol.

[107] Kailas A, Solomon JA, Mostow EN (2016). Gaps in the understanding and treatment of skin cancer in people of color. J Am Acad Dermatol.

[108] Lyons AB, Trullas C, Kohli I, Hamzavi IH, Lim HW (2021). Photoprotection beyond ultraviolet radiation: a review of tinted sunscreens. J Am Acad Dermatol.

[109] He M, Chen X, Jin S, Zhang C (2025). Visible light protection: an updated review of tinted sunscreens. Photodermatol Photoimmunol Photomed.

[110] Marionnet C, de Dormael R, Marat X (2021). Sunscreens with the new MCE filter cover the whole UV spectrum: improved UVA1 photoprotection in vitro and in a randomized controlled trial. JID Innov.

[111] Stolecka-Warzecha A, Wilczyński S, Pawlus A, Lebiedowska A, Chmielewski Ł, Niezgoda Z (2023). The use of hemispheric directional reflectance method to verify the usefulness of filters protecting the skin against infrared radiation. Clin Cosmet Investig Dermatol.

[112] Turner CW, Torgerson L (2025). Modernizing U.S. sunscreen regulations: how newer filters can improve public health. Photodermatol Photoimmunol Photomed.

[113] Flieger J, Raszewska-Famielec M, Radzikowska-Büchner E, Flieger W (2024). Skin protection by carotenoid pigments. Int J Mol Sci.

[114] Liu Y, Liu Y, Deng J, Wu X, He W, Mu X, Nie X (2022). Molecular mechanisms of marine-derived natural compounds as photoprotective strategies. Int Immunopharmacol.

